# Effect of Cold Diet and Diet at Room Temperature on Post-Tonsillectomy Pain in Children

**Published:** 2019-03

**Authors:** Mojtaba Meybodian, Mohammadhossein Dadgarnia, Mohammadhossein Baradaranfar, Sedighe Vaziribozorg, Mahzad Mansourimanesh, Mohammad Mandegari, Nasir Saeidi Eslami

**Affiliations:** 1 *Department of Otolaryngology- Head and Neck Surgery, Otorhinolaryngology Research Center, Shahid Sadoughi* * University of Medical Sciences, Yazd, Iran.*; 2 *Department of Anesthesiology* *, Shahid Sadoughi University of Medical Sciences, Yazd, Iran.*

**Keywords:** Diet, Pain, Tonsillectomy

## Abstract

**Introduction:**

The present study aimed to compare the effect of cold diet and diet at room temperature on post-tonsillectomy pain in children.

**Materials and Methods:**

In the present study a total of 120 children within the age range of 4-12 years who underwent tonsillectomy were randomly assigned to two groups, namely group C with a cold-served diet and group room temperaturewith a room-temperature-served diet postoperatively. Each patient’s post-operative pain was evaluated using the Face, Legs, Activity, Cry, Consolability (FLACC) scale prior to oral diet initiation after the operation, before thesecond acetaminophen dose, before the next day breakfast, and before discharge.

**Results:**

Out of 103 children, 48 and 55 children were femaleand male, respectively. The average age of the children was 7 years and 2 months. There was no significant difference in gender and age between the two groups. There were no significant differences in the mean scores of FLACC scale between the two groups at different times, including before starting an oral diet (P>0.15), before the second dose of acetaminophen (P>0.22), before the next day breakfast (P>0.32), and before discharge (P>0.83). In terms of bleedingfrequency, as well as nausea and vomiting, no significant difference was observed between the two groups.

**Conclusion:**

The obtained results of this study indicated that using cold liquids and foods after tonsillectomy did not have a significant effect on post-tonsillectomy pain in children. According to the findings, it is not rational to advise the mother or the child about the temperature of fluids and foods consumed post-tonsillectomy.

## Introduction

The first removal of the tonsils was reported by the Greek physician Calsus about 2000 years ago. Celsus described his surgical procedure as scratching around the tonsils, releasing, and then removing them. He used vinegar and milk for hemostasis to reduce hemorrhage (1). Tonsillectomy is still the most commonly performed surgical procedure for children in the United States with more than 530,000 procedures annually conducted (2). The most common indications of tonsillectomy are sleep-disordered breathing, tonsillitis, and peritonsillar abscess.

The most widely used technique to perform this operation is cold dissection method in which scalpel or surgical knife is utilized. After tonsillectomy, children should tolerate sore throats for one to two weeks and mostly experience referred pain in the ear. Acetaminophen is usually prescribed in order to controlpain (3). The administration of steroids can also reduce pain and nausea. 

Currently, a single dose of dexamethasone is recommended during the surgery (2). The pain post-tonsillectomy can lead to a decrease in fluid and food intake, followed by dehydration, which can slow down the repair process and make pain control harder. Different groups of analgesics have their own side effects. Therefore, the consideration of non-pharmacological ways to control pain can be of great value.

The most frequent non-pharmacological strategies used to manage pain include distraction (cognitive behavioral method) and having cold liquids/foods (ice cream) by mouth (4,5). However, in the present study and others, several patients stated that eating ice cream or cold drinks is unbearable post-tonsillectomy(6). Considering the limited number of investigations regarding the role of cold diet in the reduction of post-tonsillectomy pain, as well as the lack of an interventional, randomized, and controlled trial addressing this issue, it was decided to design and conduct such a study. The present study investigated the effect of dietary temperature on post-tonsillectomy pain.

## Materials and Methods

A total of 120 children within the age range of 4-12 years who underwent tonsillectomy were enrolled in this single-blinded study after being approved by the Ethics Committee and obtaining written informed consent. The exclusion criteria were children withcraniofacial anomalies, Down syndrome, renal and liver diseases, diabetes, asthma, preoperative sore throat, allergy to dairy products, and analgesics usage 24 h prior to surgery. 

Tonsillectomies were performed by a resident in Ear, Nose, and Throat (ENT) ward using cold dissection technique and hemostasis was achieved by cautery and suture. Following the surgery, the children were randomly assigned to two groups, including water, juice, and porridge served either cold (group C) or at room temperature (group RT). The randomization was based on patients’ rooms; therefore, no children in the same room had a different diet. The cases who did not have nausea received oral acetaminophen (10 mg/kg/dose) every 4 h. In case of a child's refusal or nausea, acetaminophen was rectally or intravenously administered. The postoperative pain was assessed in each child using the Face, Legs, Activity, Cry, Consolability (FLACC) scale at four different time points, including before oral diet(7), before the second acetaminophen dose, before breakfast of the next day, and before discharge (Table.1).

**Table 1:** Face, Legs, Activity, Cry, Consolability scale

Face	0: No particular expression or smile
	1: Occasional grimace or frown, withdrawn, disinterested
	2: Frequent constant frown, clenched jaw, quivering chin
Legs	0: Normal position or relaxed
	1: Uneasy, restless, tense
	2: Kicking or drawn up legs
Activity	0: Lying quietly, moving normal position easily
	1: Squirming, shifting back and forth, tense
	2: Arched, rigid, or jerking
Cry	0: No cry (awake or asleep)
	1: Moans or whimpers, occasional complaint
	2: Crying steadily, screams or sobs, frequent complaints
Consolability	0: Content, relaxed
	1: Reassured by occasional touching, hugging, or being talked to, distractible
	2: Difficult to console or comfort

In addition, the incidence of nausea, vomiting, bleeding, and the time of returning to a normal diet was recorded by a resident who was unaware of the child's diet. All the data were analyzed by SPSS software (version 19). P-value less than 0.05 was considered statistically significant.

## Results

A total of 120 children within the age range of 4-12 years undergoing tonsillectomy were enrolled in this single-blinded clinical trial. Out of 120 subjects, 10 children in group C and 7 children in group RT were excluded due to the lack of compliance with the diet, taking their analgesic, or the impossibility of obtaining theirinformation after discharge. Therefore, the data related to 50 children in group C and 53 children in group RT were analyzed. The participantsconsisted of 55 boys and 48 girls with an average age of 7 years and 2 months (age range: 4-12 years). 

The baseline characteristics of the children in the two groups were compared and no significant difference was observed. The FLACC scale score was calculated four times post-tonsillectomy. There were no significant differences in the mean scores of FLACC scale between the two groups at different times before starting oral diet (P>0.15), before the second dose of acetaminophen (P>0.22), before the next day breakfast (P>0.32), and before discharge (P>0.83)(Table.2).

**Table 2:** Mean scores of Face, Legs, Activity, Cry, Consolability scalebetween two groups

**Time**	**Cold Group**	**Room Temperature Group**	**P-value**
Before starting oral diet	1.35±2.88	1.45±3.28	P>0.15
Beforesecond dose of acetaminophen	1.0 ±1.71	1.21±2.00	P>0.22
Before next day breakfast	1.11±1.80	1.06±2.04	P>0.32
Beforedischarge	0.69±1.18	0.72±1.17	P>0.83

Among all, threesubjects (2.9%) had postoperative bleeding from whom one and two participants were in group C and group RT, respectively. Moreover, ten children (9.7%) had postoperative nausea and vomiting from whom six (12%) and four children (7.5%) were in group C and group RT, respectively. In terms of bleeding frequency (P>0.59), as well as nausea and vomiting (P>0.45), no significant difference was observed between the two groups. The mean scores of time for resuming normal diet in all subjects was 7.2±2.1 days following the surgery (7.4±1.8 days in group C and 7.1±2.2 days in group RT). 

The obtained results were analyzed using t-test and no significant difference was observed between the two groups (P>0.33). The mean score of required acetaminophen dose in the participants was reported as 11±2.6 mg (10.6±2.7 mg in group RT and 11.4±2.5 mg in group C). Therewas no significant difference between the two groups regarding the mean score of required acetaminophen dose (P>0.12).

## Discussion

Multiple complications can occur during or following tonsillectomy. The most common complication, that is probably a part of the natural recovery process, is pain and odynophagia. Extensive studies on various aspects of tonsillectomy have been carried out with regard to postoperative pain control. Based on the results of clinical trials addressing post-tonsillectomy pain control, several suggestions have been proposed which include using new devices in the operation, administration of analgesics, steroids or antibiotics, or simple measures, such as intravenous hydration or dietary recommendations. 

The number of studiesregarding non-pharmacological pain management post-tonsillectomy is limited. The temperature of food is one of the issues that has always been considered in the determination of children’s diet following tonsillectomy. Ice cream and cold beverages have been specifically mentioned in the instructions of surgeons post-tonsillectomy. However, there is no robust evidence behind these instructions. The aim of this study was to evaluate the effect of the temperature of liquids and foods consumed after tonsillectomy on postoperative pain in children. 

According to Otorhinolaryngology ward routine, patients who underwent tonsillectomy were hospitalized for one night after the operation. This interim allowed the authors to consistently assess and record the children’s pain based on FLACCscale at different time points. The FLACC scale was used in a series of clinical trials focusing on pain after tonsillectomy. The scoreswere originally defined for the children up to seven years of age; however, they have recently been validated for the children up to the age of 16 (8). The FLACC scale designed by Redmann et al. is considered a useful tool in measuring postoperative pain in children (9). 

The FLACC score showed a decreasing pattern over a period of 23 h after the operation. In a study carried out by Vons et al., FLACC was used to measure the children’s pain during the early hours after guillotine tonsillectomy. The maximum score calculated in their study was 7 that was recorded in the first hour following the surgery. Vons et al. continued to measure the FLACC score until the children were discharged. The lowest scores were obtainedat the patient's discharge time (10). 

In the present study, the FLACC scores in both groups were continuously decreased during the four measurement times thatis consistent with the results of the aforementioned study. Since many tonsillectomy patients in our center were from areas that could not speak Farsi, it was sometimes difficult to communicate with them. It is not efficient to communicate with young children who in addition to having anxiety due to hospital admission, experience postoperative pain. 

The FLACC scale is an objective method; therefore its value cannot be overemphasizedregarding the assessment of pain in children with difficulties in communication. Hemorrhage is the most common severe complication after tonsillectomy, occurring in 1-10% of the patients. Early bleeding following tonsillectomy occurs in the first 24 h after the surgery and is highly related to the surgical technique, while delayed bleeding over the first ten days is due to eschar sloughing from the tonsillar bed (3). 

All three cases with bleeding in the present study were delayed, and there was no significant difference between the studied groups concerning the incidenceof bleeding. The effectiveness of hot water irrigation for controlling posterior nasal epistaxis has been acknowledged by several studies (11,12). Recently, the effects of packing with gauze soaked in warm saline after tooth extraction have been investigated and advised (13).Therefore, the assumption that post-tonsillectomy cold diet can reduce the risk of hemorrhage is in contrastwiththe abovementioned evidence in controlling epistaxis and bleeding following tooth extraction. 

The time interval between tonsillectomy and the resumption of routine diet is one of the important variables measured in studies that compared various techniques of tonsillectomy and methods of pain control in patients post-tonsillectomy. The shorter time interval is an advantage for surgical or interventional therapies to reduce pain. In this study, the mean values of time to resume routine diet in group C and group RT were reported as 7.4±1.9 and 7.2±2.1 days, respectively, which was not significantly different. 

The above-mentioned period was not considerably different from those in the studies that used the same surgical procedure as the present study. For example, in a study carried out by McGregor et al., this interval was 7 daysand in studies conducted by Temple and Tim, it was 7.6 days (14, 15). Many surgeons believe that the amount of food consumed after tonsillectomy is associated with a reduction in pain. Since ice cream contains high levels of simple carbohydrates and physiologically suppresses appetite, its consumption may reduce appetite and subsequently decrease the child's desire to eat other foods and create a vicious cycle to exacerbate the pain. 

In a study conducted by Sylvester, ice lollies were given to children immediately after tonsillectomy. The pain was measured at the intervals of 15, 30, and 60 min, as well as 4 hpostoperatively. The results of their study showed that in the 30th and 60th min after the surgery, children who had ice lollies experienced less pain than controls(16). Given the fact that in the present study the intervention was delayed, compared to that in Sylvester’ study, the lack of difference between the case and control groups in this study can be attributed to the difference in the onset of the intervention and the time of pain measurement. 

Sutters et al.concluded that the most frequent non-pharmacological strategies used to manage pain include distraction and having cold liquids and foods after tonsillectomy in children (5). In the present study, no control or intervention division has been used and the data were obtained through interviewing the children with the retrospective experience of pain control methods. Coldsaline irrigation, the use of coolant devices in the tonsillar fossa, and cold compress on extracted tooth site showed the beneficial role of cryotherapy in the reductionof postoperative pain(17-20). 

In all the aforementioned instances, the interventions were performed during the surgery. When the results of the aforementioned studies are compared with the findings of the present study, it seems that cryotherapy for postoperative pain management should be applied during the surgery not several hours after that.

## Conclusion

The subjects who had cold diet post-tonsillectomy did not experience less pain than those with diet at room temperature. Furthermore, the consumption of liquids and foods at room temperature did not lead to increase complications, such as bleeding, as well as nausea and vomiting. The number of days that elapsed before resuming the routine diet and the number of acetaminophen dose administration did not correlate with the temperature of consumed foods and beverages. The obtained results of this study indicated that it was not reasonable to recommend a cold diet for children post-tonsillectomyand advise parents to offer cold foods after tonsillectomy because there is no scientific basis in this regard.
